# *Krox20* hindbrain regulation incorporates multiple modes of cooperation between *cis*-acting elements

**DOI:** 10.1371/journal.pgen.1006903

**Published:** 2017-07-27

**Authors:** Elodie Thierion, Johan Le Men, Samuel Collombet, Céline Hernandez, Fanny Coulpier, Patrick Torbey, Morgane Thomas-Chollier, Daan Noordermeer, Patrick Charnay, Pascale Gilardi-Hebenstreit

**Affiliations:** 1 Ecole normale supérieure, PSL Research University, CNRS, Inserm, Institut de Biologie de l’Ecole normale supérieure (IBENS), Paris, France; 2 Sorbonne Universités, UPMC Univ Paris 06, IFD, Paris, France; 3 Institute for Integrative Biology of the Cell (I2BC), CEA, CNRS, Université Paris-Sud, University Paris-Saclay, Gif-sur-Yvette, France; Centro Andaluz de Biología del Desarrollo, SPAIN

## Abstract

Developmental genes can harbour multiple transcriptional enhancers that act simultaneously or in succession to achieve robust and precise spatiotemporal expression. However, the mechanisms underlying cooperation between *cis*-acting elements are poorly documented, notably in vertebrates. The mouse gene *Krox20* encodes a transcription factor required for the specification of two segments (rhombomeres) of the developing hindbrain. In rhombomere 3, *Krox20* is subject to direct positive feedback governed by an autoregulatory enhancer, element A. In contrast, a second enhancer, element C, distant by 70 kb, is active from the initiation of transcription independent of the presence of the KROX20 protein. Here, using both enhancer knock-outs and investigations of chromatin organisation, we show that element C possesses a dual activity: besides its classical enhancer function, it is also permanently required in *cis* to potentiate the autoregulatory activity of element A, by increasing its chromatin accessibility. This work uncovers a novel, asymmetrical, long-range mode of cooperation between *cis*-acting elements that might be essential to avoid promiscuous activation of positive autoregulatory elements.

## Author summary

The formation of multicellular organisms from the egg to the adult stage is largely under genetic control. The activation of specific genes is governed by regulatory DNA sequences present nearby on the chromosome. Most of these sequences promote activation and are called enhancers. In this paper, we study two enhancers governing the expression of a gene involved in the formation of the posterior brain in vertebrates. One of these enhancers is involved in a positive feedback loop: it is itself activated by the protein product of the gene that it regulates. The other enhancer was thought to be only involved in the initial accumulation of the protein, necessary for the subsequent activation of the feedback loop. Here we show that the second enhancer directly cooperates with the autoregulatory enhancer to increase its accessibility and its activity. Our work uncovers a novel, long-range mode of cooperation between enhancers that restricts the domain of action of autoregulatory enhancers within embryos and might be essential to avoid their inappropriate activation.

## Introduction

DNA *cis*-acting elements play key roles in the regulation and evolution of gene expression by controlling spatiotemporal transcription patterns. A major class of *cis*-regulatory elements are transcriptional enhancers, which can recruit combinations of transcription factors (TFs) to modulate transcription initiation from (a) cognate gene promoter(s), in general independently of their relative distance and orientation [1–3]. So far, most enhancers have been functionally characterized by assay of their transcriptional activity using transgenic constructs carrying the enhancer and a reporter gene driven by a minimal promoter [4]. Another strategy consists in the random insertion of a transposon that senses enhancer activity within the surrounding genomic region. It is particularly useful to detect multiple *cis*-regulatory elements with similar activities and long-distance modulation of gene expression [5,6]. Transgenesis using BACs allows the introduction of large DNA fragments containing enhancers in their native context. This approach is helpful in the analysis of multiple enhancers controlling the same gene [7], but can be challenging for the study of mammalian enhancer that are located far away from the promoter that they control. These different approaches provide useful information on spatial and temporal activity of the putative enhancer element, but they usually do not establish whether and how the enhancer actually participates in the control of the expression of its suspected cognate gene in its full normal genomic context. Answer to this latter question requires *in vivo* analyses involving deletion or mutation of the endogenous enhancer. This issue is particularly important in situations where multiple, overlapping enhancers operate within the same *cis*-regulatory landscape. In such cases, various types of regulatory crosstalk can occur between the enhancers, resulting in additive, synergistic, competitive or repressive effects [3]. In vertebrates, very few studies have addressed such situations.

Enhancer activity is intimately linked to chromatin organization. Hence, association of pioneer TFs to an enhancer can lead to chromatin decompaction and facilitate the binding of additional TFs and/or recruitment of various epigenetic machineries [8]. In return, chromatin configuration can affect gene expression by modulating long-range interactions between enhancers and promoters [9], that are usually constrained within regions called topologically associated domains (TADs) [10,11]. TADs, which are approximately Mb-sized in mammals, form constitutive “regulatory neighbourhoods” that provide specificity to enhancer-promoter interactions by reducing aberrant contacts between *cis*-regulatory elements located in distinct TADs [6,10].

To provide insights into the mechanisms involved in the regulation of a vertebrate gene by multiple enhancers during development, we investigated the case of the mouse *Krox20/Egr2* gene [12] for which several hindbrain-specific enhancers have been identified [13]. The hindbrain is an attractive model to investigate the genetic control of morphogenesis in vertebrates, as it is subject to a transient segmentation process leading to the formation of 7–8 segments called rhombomeres (r) [14,15]. The formation and specification of segments r3 and r5 are governed by the transcription factor KROX20/EGR2 [15–17]. So far three evolutionarily conserved sequences exhibiting enhancer activity in the hindbrain have been identified within the *Krox20* locus and are termed A, B and C [13]. Element A, located 217 kb upstream of the promoter in the mouse, is active in both r3 and r5. This element carries several KROX20 binding sites and requires direct binding of the protein for its activity, suggesting that it acts as an autoregulatory element [13]. Indeed, upon deletion of element A, *Krox20* expression is normally initiated in r3 and r5, but is not amplified nor maintained at later stages [18]. Additional studies have indicated that element A underlies a positive feedback loop that acts as a binary switch for specification of odd- versus even–numbered rhombomere identity [18]. Element B, located 164 kb upstream of the promoter, drives the expression of reporter constructs specifically in r5 [13,19,20]. Finally, element C, located 144 kb upstream of the promoter, is active in the r3-r5 region [13,19–21]. Several observations suggest that elements B and C, in contrast to the autoregulatory element A, are involved in the initial steps of *Krox20* expression in r3 or r5 (initiator elements): i) they are transcriptionally active at the early stages of *Krox20* hindbrain expression [13]; ii) they are activated by transcription factors known to act upstream of *Krox20* [19–21]; iii) they do not require the presence of the KROX20 protein for their activity [13].

In the present study, we have investigated the contribution of element C to *Krox20* expression, as it was the only characterized initiator element with an activity in r3. Using a conditional knock-out mutation of element C, we show that, unexpectedly, this element is not necessary for *Krox20* initial expression in r3. In contrast, it appears absolutely required for the maintenance of *Krox20* expression in this rhombomere. This activity involves a cooperation in *cis* with element A, element C potentiating its activity and increasing its accessibility. These observations reveal that a *cis*-acting element can cooperate with other enhancers within the same locus according to different modalities and suggest a scheme for protecting autoregulatory elements from inappropriate activation.

## Results

### In r3, element C is required for late, but not initial *Krox20* expression

**Fig 1:**
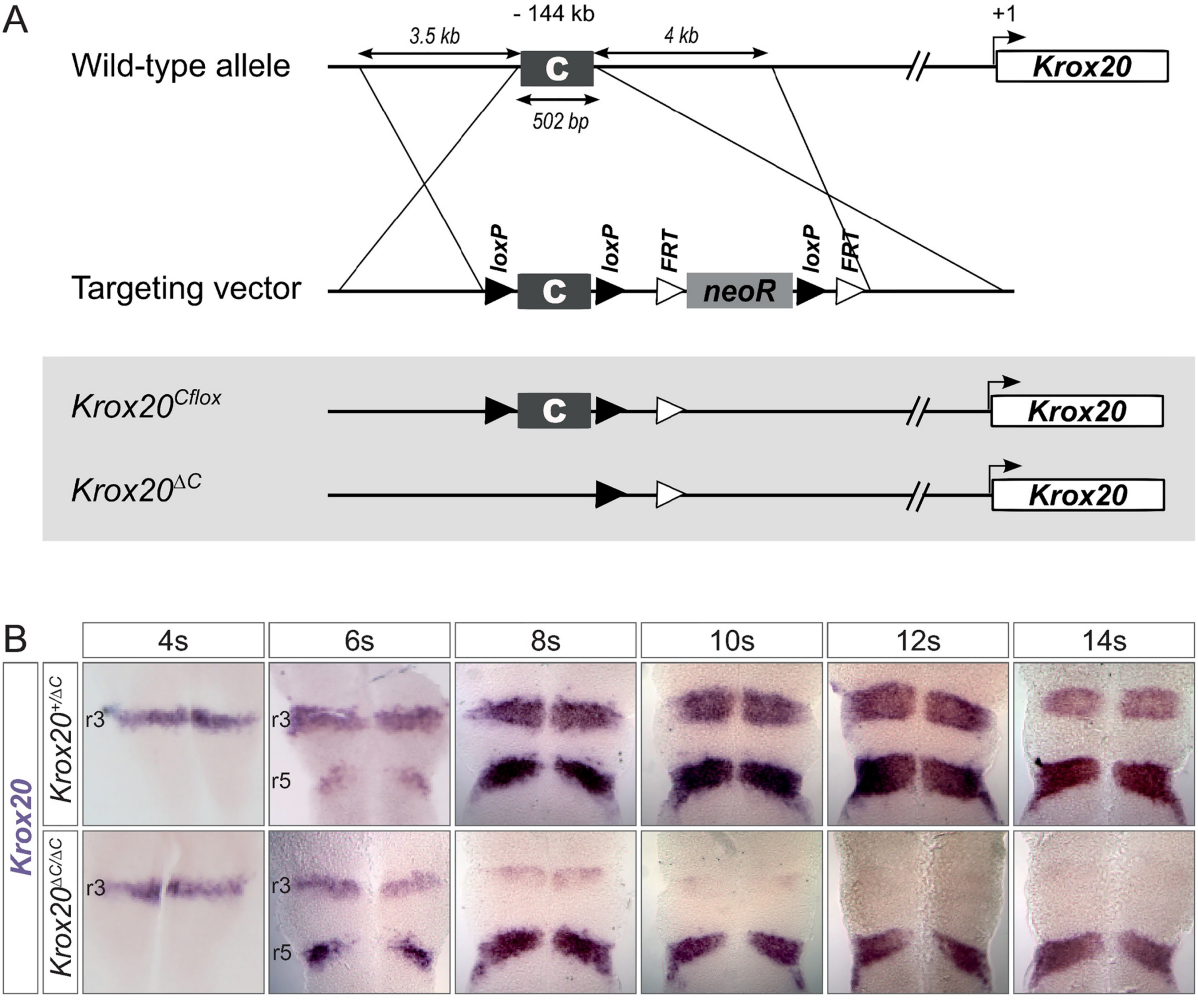
Genetic analysis of element C function. **(A)** Strategy for the construction of conditional and null alleles of element C. The targeting vector was introduced into the locus in ES cells by homologous recombination and one of the ES clones subsequently allowed germ line transmission in the mouse. The floxed allele, *Krox20*^*Cflox*^, was obtained by crossing the founder mouse line with a *Flp* (targeting FRT sites) deletor line. The null allele, *Krox20*^*ΔC*^, was obtained by crossing the *Krox20*^*Cflox*^ line with a *Cre* (targeting loxP sites) deletor line, PGK-Cre. **(B)** In situ hybridization for *Krox20* mRNA performed on *Krox20*^*+/ΔC*^ and *Krox20*^*ΔC/ΔC*^ embryos at the indicated somite stages. Embryos were flat-mounted with anterior toward the top. Rhombomere positions are indicated on the left.

To assess the contribution of element C to the regulation of *Krox20* expression, we generated a mouse line carrying a deletion of this element. The details of the knock-out strategy are presented in Fig 1A. Two alleles were generated: *Krox20*^*Cflox*^, in which element C is present, but flanked by two loxP sites, and *Krox20*^*ΔC*^, in which element C is deleted. The impact of element C deletion on *Krox20* transcription was analysed by mRNA *in situ* hybridization on 4 to 14 somite stage (s) embryos, comparing homozygous (*Krox20*^*ΔC/ΔC*^) with heterozygous mutants (*Krox20*^*+/ΔC*^), the knock-out of one allele of *Krox20* having no phenotype [15,16,22]. Unexpectedly, up until 6s the expression of *Krox20* in r3 and r5 is similar in *Krox20*^*ΔC/ΔC*^ embryos and control littermates (Fig 1B). However, at 8s, *Krox20* expression is severely reduced in r3 from *Krox20*^*ΔC/ΔC*^ embryos as compared to controls and, at later stages, it is completely lost (Fig 1B). During the considered period, although *Krox20* expression does not appear to be dramatically affected in r5, in contrast to r3, the width of the corresponding domain of expression appears to be slightly reduced after 10s (Fig 1B).

To investigate longer-term consequences of element C deletion on cell specification, we analysed the expression of a KROX20 target gene, *EphA4* [22], which is known to persist beyond the period of *Krox20* expression in r3 and r5 [18]. In control embryos (*Krox20*^*+/ΔC*^), at 10s and 25s, *EphA4* is expressed at high levels in r3 and r5 and at a lower level in r2 (S1 Fig). At both stages, the r3 domain, as demarcated by *EphA4* expression, is reduced in *Krox20*^*ΔC/ΔC*^ embryos as compared to controls, whereas the r5 domain is similar in both genotypes (S1 Fig). This is consistent with the premature loss of *Krox20* expression in r3, known to reduce the extension of this rhombomere [18,22]. The limited variation of *Krox2*0 expression in r5 in *Krox20*^*ΔC/ΔC*^ embryos after 10s does not appear to perturb the size of this rhombomere at later stages, consistent with the fact that *Krox20* expression is not required for the maintenance of *EphA4* expression in r3 and r5 [18].

**Fig 2:**
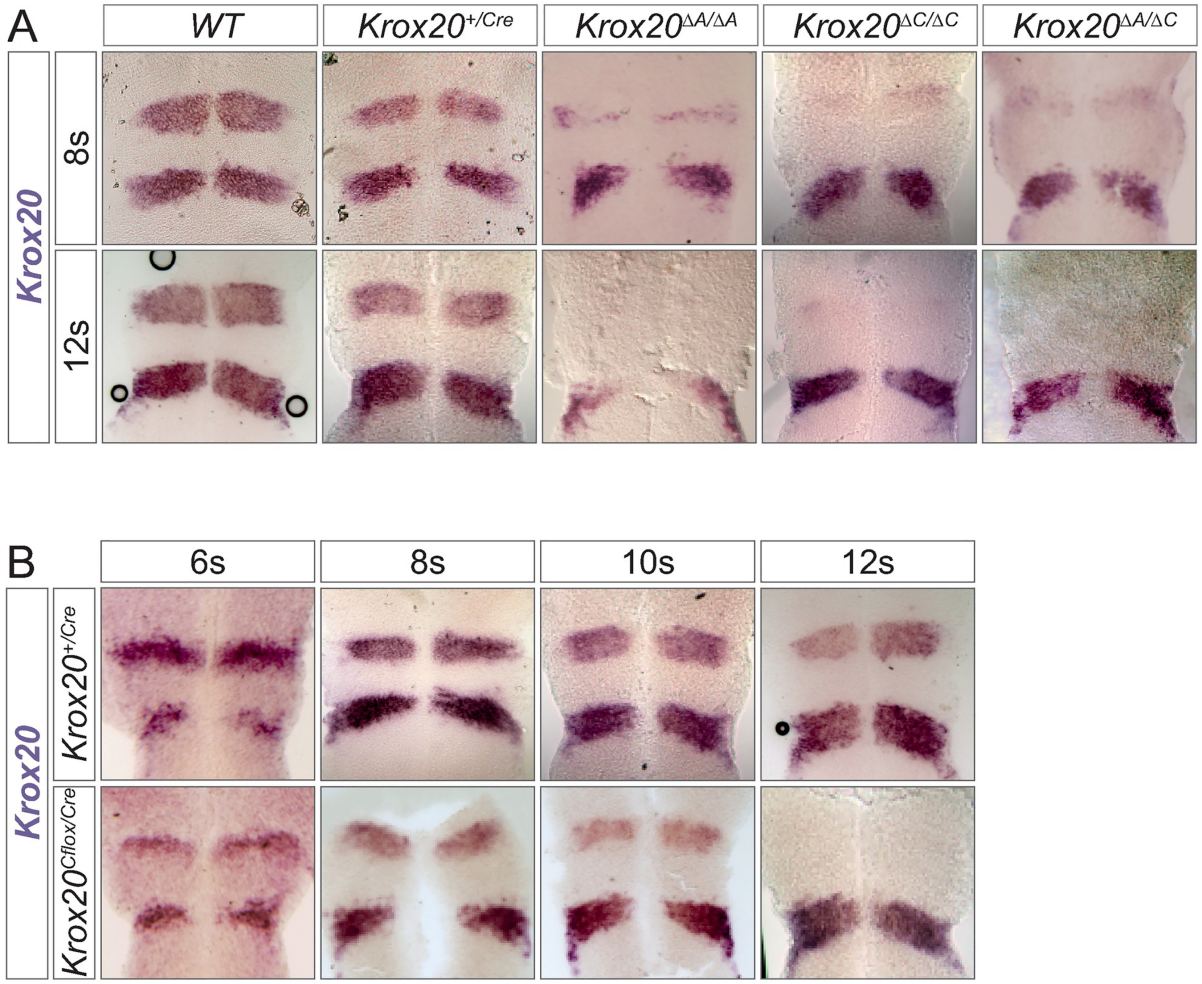
**Cooperation in *cis* between elements A and C. (A)** In situ hybridization for *Krox20* mRNA was performed on wild type (WT), *Krox20*^*+/Cre*^, *Krox20*^*ΔA/ΔA*^, *Krox20*^*ΔC/ΔC*^ and composite heterozygous *Krox20*^*ΔA/ΔC*^ embryos at the indicated somite stages. **(B)** In situ hybridization for *Krox20* mRNA was performed on *Krox20*^*+/Cre*^ and *Krox20*^*Cflox/Cre*^ embryos at the indicated somite stages. In (A) and (B) embryos were flat-mounted with anterior toward the top.

In conclusion, these data indicate that i) element C is dispensable for the initiation of *Krox20* expression in r3 or r5, suggesting the existence of other elements in charge of these functions; ii) in r3, in contrast, element C is absolutely required for expression beyond 6s, leading to a reduction in size of this rhombomere at later stages. Notably, the phenotype observed in r3 in *Krox20*^*ΔC/ΔC*^ embryos is very similar to what was previously described in *Krox20*^*ΔA/ΔA*^ embryos (Figs 1B and 2A, and S1 Fig) [18]; iii) in r5, the contribution of element C to *Krox20* expression is rather limited, without significant effect on the size of this rhombomere.

### Elements A and C cooperate in *cis* for the establishment of the autoregulatory loop

The similarity of the phenotypes observed in r3 upon deletion of elements A or C led us to investigate the possibility of an involvement of element C in *Krox20* autoregulation, together with element A. For this purpose, we first analysed the expression of *Krox20* in composite heterozygous embryos, *Krox20*^*ΔA/Δ*C^, carrying deletions of element A on one allele and of element C on the other (Fig 2A). Although this combination does not affect *Krox20* expression in r3 at early stages, at 8s *Krox20* mRNA level is severely reduced and, at 12s, it is completely lost, mimicking the phenotype observed in *Krox20*^*ΔA/ΔA*^ or *Krox20*^*ΔC/ΔC*^ embryos at both stages (Fig 2A). In r5, *Krox20* expression is only slightly affected in *Krox20*^*ΔA/Δ*C^ embryos, similarly to *Krox20*^*ΔC/ΔC*^ embryos (Fig 2A). This defect in the maintenance of *Krox20* expression in r3, combined with apparently normal expression at early stages, contrasts with the fact that a single wild type *Krox20* allele is sufficient to activate and maintain the autoregulatory loop (Fig 2A, *Krox20*^*+/Cre*^) [12]. This suggests that in *Krox20*^*ΔA/Δ*C^ embryos the level of expression of *Krox20* is not a limiting factor for the activation of the only wild type allele of element A. Therefore, the most likely explanation for the defect in *Krox20* maintenance is that, in r3, the deletion of element C impairs the activity of element A located on the same chromosome and that the two elements synergistically cooperate, in *cis*, for the establishment and/or maintenance of the autoregulatory loop. A more conventional, partial redundancy between elements A and C appears much less likely.

This cooperation does not preclude an early involvement of element C, for instance to poise element A for the subsequent autoregulation phase. To investigate whether element C has a function only at the early phase of *Krox20* activation, or whether it is required during the autoregulation phase as well, we generated a genetic condition in which element C is initially active, but is deleted at a later stage. This was achieved by combining the *Krox20*^*Cflox*^ allele (Fig 1A) with a knock-in allele, *Krox20*^*Cre*^, in which the *Krox20* coding sequence has been replaced by the coding sequence of the Cre recombinase [23]. In such embryos, *Krox20* and *Cre* are expected to be synthetized at early somitic stages. Subsequently, the recombinase leads to deletion of element C in r3 and r5. In *Krox20*^*Cflox/Cre*^ embryos, *Krox20* expression is progressively reduced in r3 from 6s to 10s, as compared to *Krox20*^*+/Cre*^ controls (Fig 2B), although less abruptly than in *Krox20*^*ΔC/ΔC*^ mutants (Figs 1B and 2A). At 12s, *Krox20* expression is completely abolished in r3 in *Krox20*^*Cflox/Cre*^ embryos (Fig 2B). These data indicate that the presence of element C only during the early phase of *Krox20* expression is not sufficient to establish and/or maintain the autoregulatory loop. The higher level of *Krox20* in r3 in *Krox20*^*Cflox/Cre*^ as compared to *Krox20*^*ΔC/ΔC*^ embryos is likely to originate from transient activation of the loop, followed by termination of its activity, due to Cre excision of element C.

In conclusion, these results indicate that elements A and C synergistically cooperate in *cis* for establishing and/or maintaining this loop in r3. More precisely, they show that element C is permanently required for activity of the *Krox20* feedback loop.

### Physical interactions between *Krox20 cis*-elements

**Fig 3:**
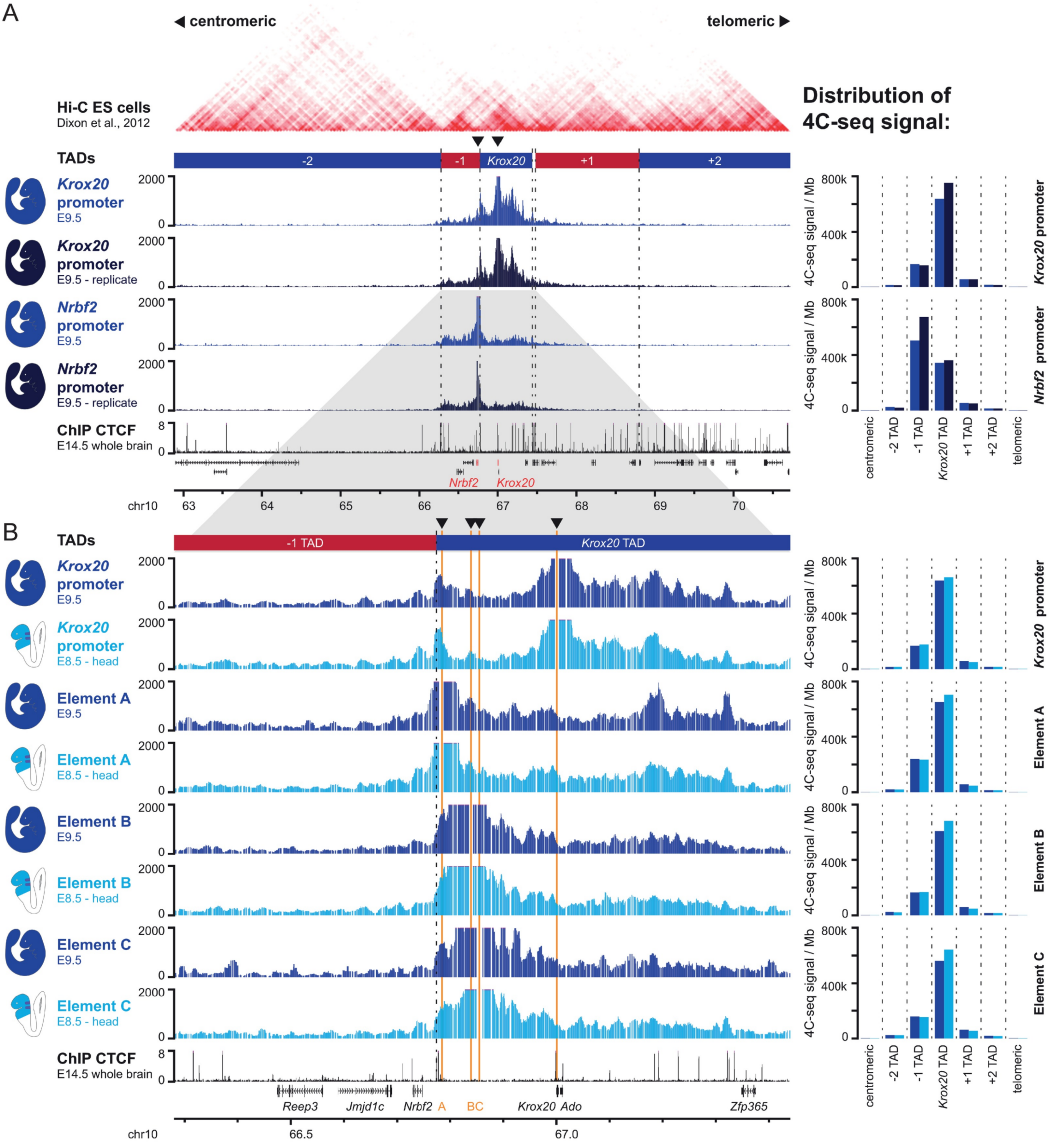
Physical interactions within the *Krox20* locus. **(A)** Alignment of data in the *Krox20* and adjacent loci from Hi-C in ES cells [11], 4C-seq in E9.5 whole mouse embryos, using the *Krox20* and *Nrbf2* promoters as viewpoints (this work, 2 biological replicates) and CTCF ChIP-seq in E14.5 mouse brain (ENCODE, [58]). **(B)** Zoom in on the *Krox20* locus, showing 4C-seq data from the *Krox20* promoter, element A, element B and element C as viewpoints. CTCF ChIP-seq data in E14.5 mouse brain (ENCODE) are indicated below. Signals from simultaneously processed E9.5 whole embryo (dark blue) and E8.5 embryo head (light blue) samples are shown. On the right, normalized distributions of the 4C-seq signals in different genomic regions are indicated. TADs as defined in [7] or by our additional analysis (S2 Fig) are indicated above, with dashed lines in the graphs demarcating TAD boundaries. Genes (black/red), *cis*-regulatory elements (orange) and genomic coordinates are indicated below each set of data. Arrowheads above each 4C track pinpoint viewpoints.

The existence of a cooperation in *cis* between elements A and C led us to investigate the existence of possible physical 3D interactions between the different *Krox20 cis*-elements, which are separated by large distances on the mouse chromosome. A previous Hi-C analysis [11] in embryonic stem cells identified a TAD that includes the gene and its *cis*-regulatory elements (Fig 3A). The left boundary of the TAD spreads out over a relatively large and undefined transition zone (S2A and S2B Fig). To better characterize the *Krox20* regulatory neighborhood, we used circular chromosome conformation capture (4C-seq) on multiple viewpoints in the locus [24]. In samples prepared from total embryos at embryonic day (E) 9.5, when *Krox20* is no more transcribed [25], the *Krox20* gene and its distant regulatory element A (separated by over 200 kb) show highly similar distributions of 4C-seq signal (Fig 3A and S2B and S2C Fig) preferentially located in the *Krox20* TAD. In contrast, the distribution of the *Nrbf2* gene, which is located in the TAD transition zone and is separated from element A by a 35 kb genomic interval (including a cluster of CTCF binding sites) spreads its interactions about equally over the two neighboring TADs (S2C Fig). Repositioning of the TAD boundary to the cluster of CTCF binding sites results in strongly increased separation of signal between the *Nrbf2* gene on one hand and the *Krox20* gene and its regulatory elements on the other hand, indicating they are located in different regulatory neighborhood (Fig 3A and S2C Fig).

To determine if 3D chromatin interactions in the *Krox20* regulatory neighborhood were dynamic at these early stages of embryogenesis, and possibly linked to the autoregulatory loop, we compared our E9.5 samples to micro-dissected embryonic heads at E8.5, when the autoregulatory loop is active in a subset of cells [18]. For all viewpoints, very similar patterns were obtained between E8.5 heads and E9.5 (Fig 3B). At both stages, the *Krox20* promoter forms long-range interactions within the *Krox20* TAD that cover elements A and B (Fig 3B). In addition, bi-directional interactions are formed between elements A and B and, to a lesser extent, between elements A and C (Fig 3B).

In conclusion, these data reveal that the *Krox20* regulatory neighbourhood adopts a higher-order configuration that incorporates long-range interactions between the various *cis*-regulatory elements and is mostly invariant at different positions in the early embryo.

### Chromatin accessibility of *Krox20* enhancers correlates with transcriptional activity

**Fig 4:**
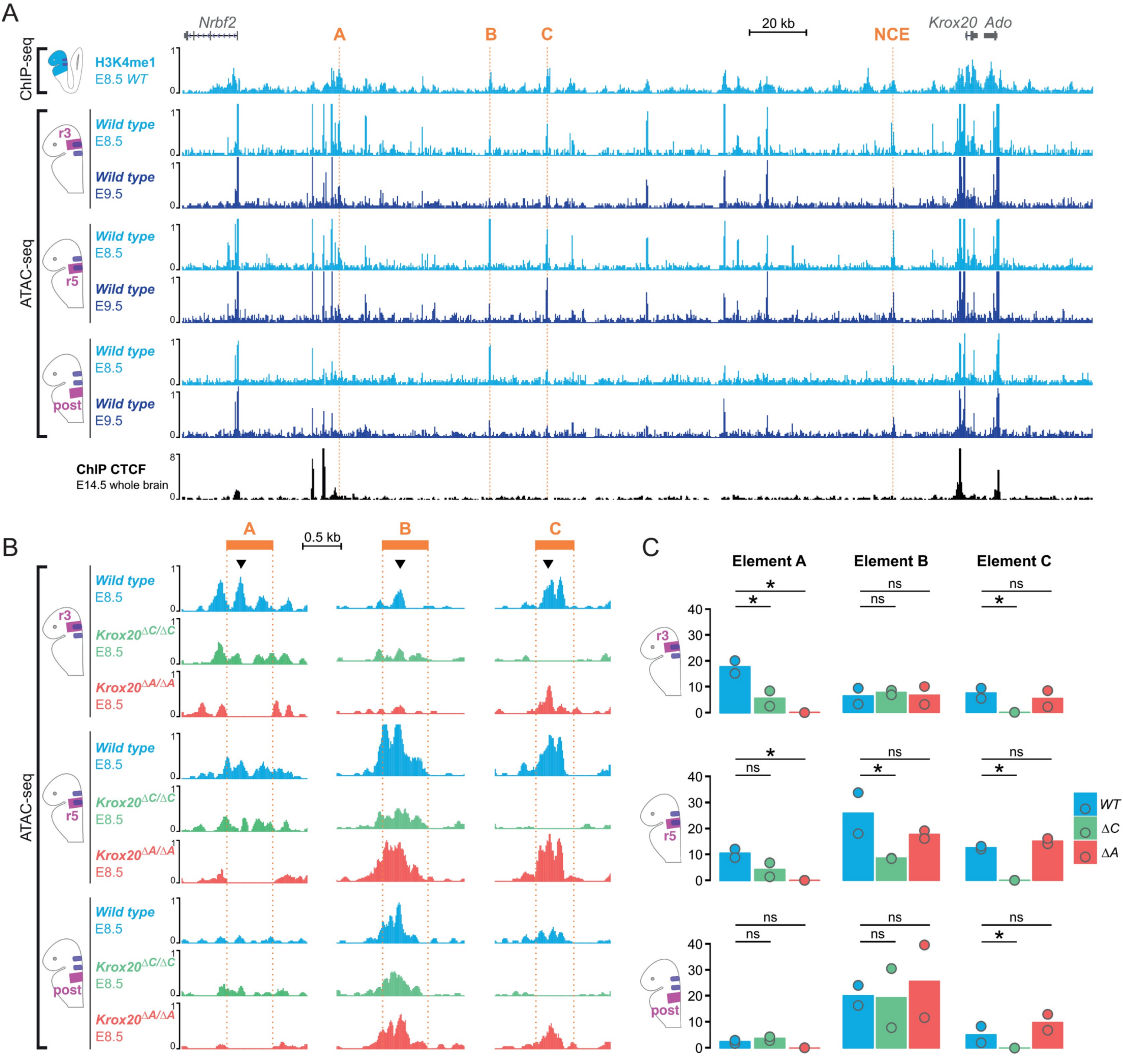
Chromatin modifications and accessibility within the *Krox20* locus. **(A)** ChIP-seq was performed for the H3K4me1 mark on wild type E8.5 embryo heads (light blue) using biological duplicates. Only one set of data is shown. ATAC-seq was performed on dissected regions (r3, r5 and a more posterior region (“post”; see text) from wild type embryos at E8.5 (light blue) and E9.5 (dark blue) using biological duplicates. Only one set of data is shown. CTCF ChIP-seq data from E14.5 mouse brain (ENCODE) are indicated below (see Fig 3A). Genes, *cis*-regulatory elements (orange) and a genomic scale are indicated at the top. **(B)** ATAC-seq was performed on dissected parts from wild type (light blue), and *Krox20*^*ΔC/ΔC*^ (green) and *Krox20*^*ΔA/ΔA*^ (red) E8.5 embryos using biological duplicates. Only one set of data is shown. *Cis*-regulatory elements (orange) and a genomic scale are indicated at the top. Arrowheads indicate the summits (defined by macs2 after peak calling, see Material and methods) used for quantifications in (C). **(C)** Barplots showing signal intensity of ATAC-seq (normalized fragment counts) at the summit of each element (arrowheads in panel B) for wild type (WT, blue) and *Krox20*^*ΔC/ΔC*^ (ΔC, green) embryos at E8.5 for each dissected part. The statistical significance was calculated using a negative binomial Wald Test (R package DESeq2) on the 2 replicates, which are represented by dots. Star indicates p-value < 0.05. ns: non-significant.

To investigate the correlation between the activity of the *Krox20 cis*-regulatory elements and their chromatin modifications and conformation, we first performed ChIP-seq experiments [26] to analyse two histone modifications: H3K4me1 (broad peaks covering active enhancers) and H3K27ac (punctuated peaks covering active enhancers and promoters) [27]. In E8.5 wild type embryo heads, a number of H3K4me1 peaks were observed, including those that expectedly correspond to the A, B and C elements and to a previously known neural crest element (NCE; Fig 4A) [28]. The signals observed for the H3K27ac mark were low across the *Krox20* locus except for the promoter (S3A Fig). We can observe the same pattern of H3K27ac at the *EphA4* locus with low signal at the enhancer driving its expression in r3 and r5 [29] and higher signal at the promoter (S3B Fig). In contrast, a gene widely expressed at E8.5 in the whole embryo head, like *Sox2* [30], displays a high H3K27ac enrichment (S3C Fig). The low signals observed for the *Krox20* and *EphA4* genes are most likely due to the limited number of *Krox20*-expressing cells in the sample.

To overcome this limitation, we performed micro-dissections and assessed chromatin structure by ATAC-seq [31], a technique that requires much lower cell numbers (a few thousand). Enhancer activity is usually associated with increased local chromatin accessibility. E8.5 (8s-10s stage) or E9.5 embryos were dissected to isolate samples derived from three regions: an anterior region, obtained by cutting within r2 and r4 (r3 sample); an intermediate region, for which cutting was performed within r4 and r6 (r5 sample), and a posterior region, for which cutting was performed within r6 and r7-8 (posterior sample). We observed peaks of accessibility at the level of the promoter at both stages and in all of the regional samples (Fig 4A). At both stages, element A was specifically accessible in the r3 and r5 samples, but not in the posterior sample (Fig 4A and 4B), in accordance with its activity restricted to r3 and r5 [13]. At E8.5, element B was compacted in the r3 sample, but highly accessible in the r5 and posterior samples (Fig 4A and 4B). This accessibility largely decreased at E9.5 (Fig 4A). The limited accessibility of element B in r3 is in agreement with its lack of activity in this rhombomere [13]. Finally, element C was particularly accessible in the r3 and r5 samples at E8.5 (Fig 4A and 4B), consistent with its activity that spans the r3-r5 region [13]. This accessibility was only maintained in the r5 sample at E9.5 (Fig 4A).

The pattern of chromatin accessibility observed in our ATAC-seq experiments revealed additional potential enhancers involved in the regulation of the *Krox20* gene in the hindbrain. Indeed, we have identified an element located 107 kb downstream to *Krox20* with high accessibility at E8.5 (S3A Fig). We have tested the transcriptional activity of this new element (NE) by transgenesis in the zebrafish by cloning it upstream of a *GFP* reporter gene. In a transgenic line, this element drives specific expression in r3 at the time of the initiation of *Krox20* expression in this rhombomere (S4 Fig). These data raise the possibility that element NE might be the missing element involved in the initiation of *Krox20* expression in r3, although its activity still needs to be verified in the mouse.

In conclusion, this analysis reveals that the patterns of accessibility of the different known elements largely correlate with their enhancer activities and helped us to identify a novel candidate element for the regulation of *Krox20* expression in r3.

### Element C modulates the accessibility of enhancer A

A final step was to investigate the effects of enhancer deletions on the accessibility of the other elements. Deletion of element A did not significantly affect the accessibility of elements B or C in any samples (Fig 4B and 4C and S3A Fig). In contrast, deletion of element C significantly reduced the accessibilities of element A in r3 and of element B in r5 (Fig 4B and 4C and S3A Fig). These data establish that element C has the capacity to specifically modulate the accessibility of elements A and B and therefore probably their activities. They may provide a mechanism for the involvement of the late activity of element C in the control of *Krox20* autoregulation governed by element A. Furthermore, this analysis establishes the existence of an asymmetry in the relationship between elements A and C: whereas element C affects A accessibility and presumably potentiate its activity, the reverse is not true.

## Discussion

In the present study, we have made a further step in the understanding of the molecular mechanisms governing the expression of a master developmental regulator, using both enhancer knock-outs and investigations of chromatin structure. This analysis reveals that *Krox20* regulation relies on a complex crosstalk between several *cis*-acting elements that interact simultaneously according to multiple modes (redundant/additive/synergistic, symmetric/asymmetric) to shape the pattern of expression of the gene. Among these enhancers, element C performs a dual function, as a classical enhancer and as a potentiator in *cis* of element A. We propose that this latter role may constitute a general means to prevent promiscuous activities of autoregulatory elements.

### Control of the early phase of *Krox20* expression in r3

Previous analyses had suggested a rather straightforward mode of regulation of the *Krox20* gene. Element C was responsible for the initiation of its expression in r3, whereas element B, possibly together with element C, was in charge of the initiation in r5. Subsequently, element A governed the maintenance of the expression through a positive feedback loop towards a definitive engagement into an odd-numbered rhombomere fate [13,18,19]. Knock-out analysis of element C now leads to major revisions of this scenario. Despite the early r3 enhancer activity of element C, as demonstrated by transgenic experiments in mouse and fish [13,32], the deletion of mouse element C does not affect early *Krox20* expression in r3 (Fig 1B). This suggests that another *cis*-acting element contributes to this expression. This is not element B, which is only active in r5, as revealed in transgenic experiments [13], nor element A, which is absolutely dependent on the presence of the KROX20 protein [13,18]. Therefore, another enhancer, active in r3 and not dependent on KROX20, must be involved. Indeed, the identification of the NE element, fully accessible at early time in the hindbrain and specifically active in r3, makes it an attractive candidate for being involved in the initiation of *Krox20* expression in this rhombomere (S3A Fig and S4 Fig).

The absence of phenotype in *Krox20*^*ΔC/ΔC*^ embryos during the early phase of *Krox20* expression does not preclude a role for element C during this phase. In support of this idea, enhancer activity of element C in r3 is dependent on the binding of Meis and Hox/Pbx factors [21], as well as of the Sp5 factor mediating FGF signalling [19,20], factors that are precisely known to act upstream of *Krox20* in r3 [33–39]. It is therefore possible that elements C and NE cooperate in a redundant manner (S4 Fig) and further analyses will be required to determine whether this is indeed the case. Several examples of redundancy have been reported for enhancers governing the expression of developmental genes [3,40–42]. Redundant enhancers, or shadow enhancers, often share the same regulatory logic, since their activities have to be, at least in part, concomitant [43]. The analysis of the characteristics of the NE enhancer should reveal whether it depends on the same TFs as element C. In a few cases of redundant *cis*-acting elements that have been investigated in detail so far, it has been shown that redundancy provides robustness to the system and that, in specific genetic or environmental conditions, phenotypes can be revealed in absence of one of the elements [44].

### Dual function of element C

Our study also revealed an unexpected function of element C: it is required for autoregulation, which was thought to be only dependent on element A. Using genetic approaches, we showed that an interaction must occur in *cis* between the two elements and that it is permanently required during the autoregulatory phase. ATAC-seq experiments indicated that element C is likely to act by modulating the accessibility of element A. Therefore, simultaneous to its classical enhancer function, element C performs another type of activity, which we propose to name enhancer potentiator. Potentiator characteristics (asymmetrical interaction, permanent requirement, long-range) clearly distinguishes this function from classical enhancer cooperative activities (additive, synergistic) and possibly from other hierarchical logic modes of interactions [3,45].

At this point, it is not known whether this additional enhancer potentiator function of element C, which is functionally distinct from its classical enhancer activity, is dependent on enhancer activity. Several recent studies have shown that enhancers can be transcribed and that the products of this transcription can act locally in *cis* to promote the expression of the target gene [46]. It is possible that such a mechanism could be involved in the potentiator activity of element C. It is interesting to note that Nlz factors, which are likely to repress *Krox20* expression by reducing the accessibility of the KROX20 protein to element A [18], are also involved in repressing element C [32]. It is therefore possible that Nlz factors only indirectly affect accessibility of KROX20 on element A, by modulating the potentiator activity of element C.

In r5, *Krox20* is almost normally expressed in the absence of element C, suggesting that cooperation between elements A and C is not essential in this rhombomere to support element A activity. It is possible that element B, which is likely to constitute the major initiator element in r5 and physically interacts with element A (Fig 3B) performs a dual function similar to element C and potentiates the activity of element A in this rhombomere, in addition to its classical enhancer activity.

### A security lock on autoregulatory elements

Analyses by Hi-C in embryonic stem cells [11] and by 4C-seq in various embryonic samples (this report) revealed the existence of a regulatory neighbourhood that contains interactions between the *Krox20* promoter and element A, irrespective of the considered stages or regions of the embryo (Fig 3). This chromatin configuration might therefore constitute a permissive environment for the activation of the autoregulatory loop. Such a situation, in which an autoregulatory element might be only dependent on the presence of its cognate TF for its activity, would be rather dangerous for an organism, as any transcription of the TF gene, even illegitimate, could end up activating the feedback loop and lead to high-level and long-term expression of the gene. This danger would be increased by the stochastic nature of the activation of the autoregulatory loop [19]. Furthermore, developmental genes may possess several positive autoregulatory enhancers that have to function at specific stages or in different tissues. This is exemplified by the *Krox20* gene, that has at least three of such elements and that are differentially active in r3/r5, the neural crest and developing bones [13,28]. Therefore, mechanisms must exist as well to prevent the inappropriate activation of these elements in the other embryonic tissues. Indeed, we have shown that ectopic expression of exogenous *Krox20* in the entire zebrafish embryo only leads to activation of the autoregulatory loop in the r2-r6 region of the hindbrain [18].

**Fig 5:**
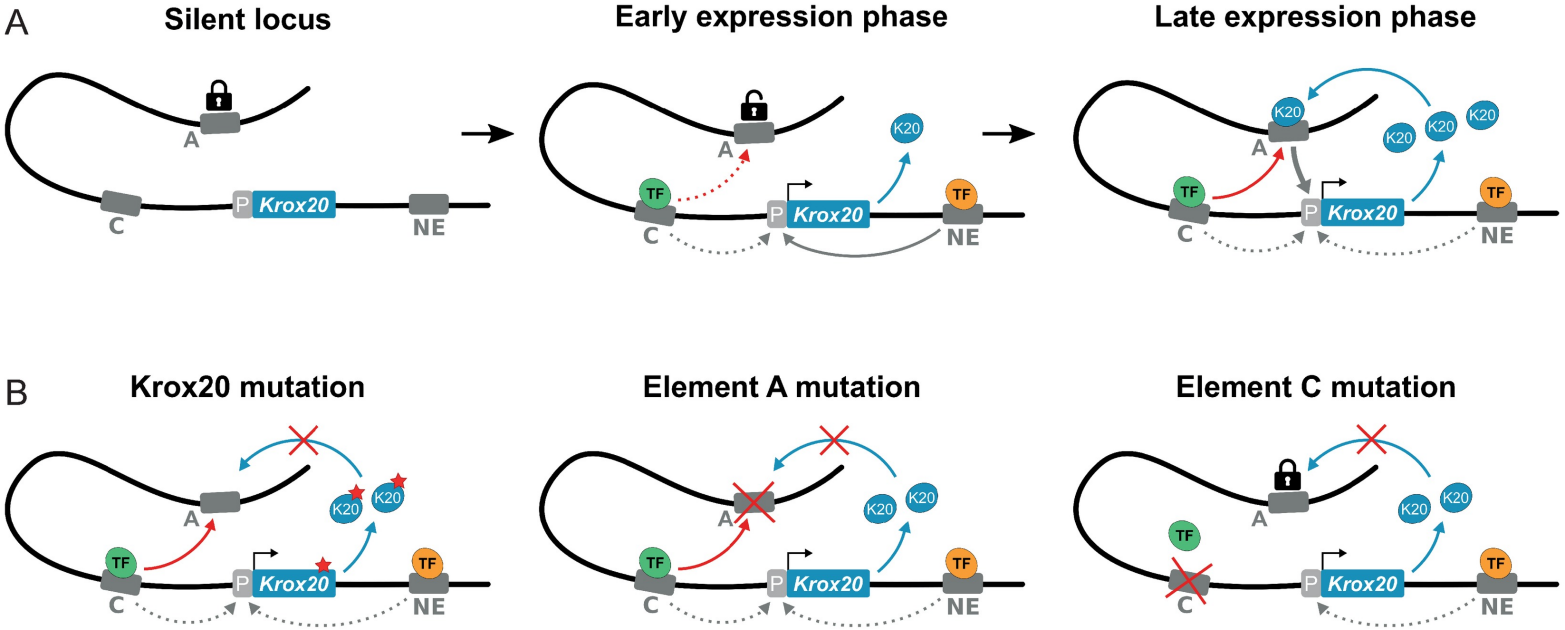
A model for *Krox20* regulation and the dual function of element C. (**A**) Schematic representation of the regulation of *Krox20* in r3. Three situations are envisaged in wild type embryos. Left: silent locus. If both element C and the new enhancer (NE) are inactive, no expression occurs. Middle: early expression phase. At this stage, elements C and NE have been bound by their respective transcription factors and have initiated the expression of *Krox20* via their classical enhancer functions. Nevertheless, element C has not yet been unlocked (decompacted) element A and/or the concentration of the KROX20 protein has not reached high enough levels to allow the establishment of a stable feedback loop with a significant probability. Right: late expression phase. Via its potentiator function, element C has unlocked element A, which can bind the KROX20 protein, which has now accumulated at a high enough concentration. Activation of enhancer A establishes the autoregulatory loop. **(B)** Three mutations that disrupt the positive feedback loop are presented at late expression phase. Left: mutation of the KROX20 protein preventing binding to element A. Middle: mutation of element A, preventing the binding of the KROX20 protein. Right: mutation of element C, preventing unlocking of element A.

The introduction of an enhancer potentiator within a positive feedback loop may constitute an efficient prevention (safety lock) against inappropriate activation of autoregulatory elements. According to our model (Fig 5A), in the ground state, the autoregulatory element (element A in the case of *Krox20*) is locked in a configuration that is not accessible to its cognate TF and therefore unable to activate transcription, despite its possible interaction with the promoter. This lock will be released when the potentiator function is provided by a second *cis*-acting element (element C). It is interesting to note that in transgenic constructs, element A is able to activate a promoter in the absence of element C *in cis*. This difference in behaviour might be explained by two types of reasons: in transgenic constructs, element A is very close to the promoter, in contrast to the endogenous enhancer, located far upstream to the promoter; the chromatin context of a transgene is likely to be different, possibly more permissive than that of a highly regulated endogenous locus.

In the endogenous locus, if the unlocker element is also responsible for the early accumulation of the cognate TF, through its classical enhancer activity, the autoregulatory element will be placed under the control of the upstream factors regulating the initial expression of the TF. In this way, the asymmetrical cooperation between the two *cis*-acting elements becomes essential for establishing the appropriate specificity of the positive feedback loop. As indicated in the model, such a feedback loop can be broken by mutation of either the TF or of any of the two *cis*-acting elements (Fig 5B).

## Materials and methods

### Ethics statement

All animal experiments were performed in accordance with the guidelines of the council of European Union directive n°2010/63/UE and were approved by the "Comité d'éthique pour l'expérimentation animale Charles Darwin" (Project Number: CE5/2012/120).

### Mouse lines and situ hybridization

All mouse lines were maintained in a mixed C57BL6/DBA2 background. We used the following alleles: *Krox20*^*Cre*^ [23] and *Krox20*^*ΔA*^ [18]; the mouse *Krox20*^*Cflox*^ line was generated at the Institut Clinique de la Souris (Illkirch, France) by homologous recombination in ES cells; the *Krox20*^*ΔC*^ allele was obtained as described in Fig 1, using the maternally expressed PGK-Cre transgene as deletor [47]. In situ hybridizations were performed on whole embryos as previously described [48], with the following digoxigenin-labelled riboprobe: *Krox20* [49] and *EphA4* [50].

### ChIP-seq

ChIP-seq experiments were performed as previously described [51]. Briefly, 10 embryos at E9.5 or 20 embryos at E8.5 were dissected in cold PBS. Cell suspensions were obtained by passing them through a 21G needle fitted onto a 5ml syringe. The cells were cross-linked with 1% formaldehyde for 10 min and washed twice in PBS, 1 mM PMSF, 1 X PIC (Protease Inhibitor Cocktail). Sonication was performed on a Covaris S220 using the following programme: duty factor = 10/5, peak incident power = 140, cycles per burst = 200 during 600/480 seconds. 5–10 μg of chromatin was used for each IP using 3 μg of the following antibodies: anti H3K4me1 (C15410037, Diagenode) and anti H3K27ac (ab4729, Abcam) in RIPA buffer. The libraries were prepared with the MicroPlex Library Preparation kit (Diagenode, E8.5 embryos) and with the NEXTflex ChIP-Seq Kit (Bioo Scientific, E9.5 embryos). ChIP Seq experiments involved biological duplicates.

Sequencing was performed on multiplexed samples using 50 bp single-end reads on an Illumina HiSeq system (E9.5 embryos) or using 42 bp paired-end reads on an Illumina NextSeq (E8.5 embryos) according to the manufacturer’s specifications. Chip-seq data were analysed as follows, using Eoulsan [52] with extended support for ChIP-seq workflows (https://github.com/GenomicParisCentre/eoulsan/tree/branch-chip-seq). First, reads were filtered out when they would not pass Illumina filters (module filterreads with option illuminaid). Files corresponding to technical replicates were merged (module technicalreplicatemerger, with option format = fastq), followed by trimming of the reads using Trim Galore! (http://www.bioinformatics.babraham.ac.uk/projects/trim_galore/; version 0.4.1 in module trimadapt, with cutadapt v1.8.1 and options: length = 41, quality = 20, error = 0.1, stringency = 8, and is.paired = yes for E8.5 data and is.paired = no for E9.5 data). Mapping was performed using STAR [53] (version 2.4.0k in module mapreads with mapper.arguments = “—outFilterMultimapNmax 1—outFilterMismatchNmax 999—outFilterMismatchNoverLmax 0.06—alignIntronMax 1—alignEndsType EndToEnd—alignMatesGapMax 2000—outSAMunmapped Within”). Further filters were applied to the mapped reads before conversion into BAM (module filtersam with removeunmapped = true; module sortsam; module rmdupgalax with is_sort = true; module sam2bam with compression.level = 5). BIGWIG files were created from the resulting BAM files using deepTools’ bamCoverage [54] (version 1.6.0, with options:—binSize 1—normalizeTo1x 200000000—fragmentLength 200—outFileFormat bigwig).

### 4C-seq

4C-seq libraries were constructed as previously described [55] with small adjustments. 35 E9.5 embryos (for each biological duplicate) or 250 E8.5 embryos were dissected in cold PBS, followed by dissociation in collagenase type I (Gibco). DpnII (New England Biolabs, Ipswich, MA) was used as the primary restriction enzyme and NlaIII (New England Biolabs) was used as the secondary restriction enzyme. For each viewpoint, up to 600 ng of each 4C-seq library were amplified using 16 individual PCR reactions with inverse primers including Illumina adapter sequences (S1 Table). Illumina sequencing was performed on multiplexed samples, containing PCR amplified material of up to 7 viewpoints, using 100 bp single-end reads on the Illumina HiSeq system according to the manufacturer's specifications at the iGE3 Genomics Platform of the University of Geneva (Switzerland). Reads were sorted, aligned, translated to restriction fragments and smoothed (11 fragments running mean) using the 4C-seq pipeline of the BBCF HTSstation [56] according to ENSEMBL Mouse assembly NCBIM37 (mm9). For the calculation of the 4C-seq signal distribution, reads were normalized to the entire chromosome 10, based on an approach adapted from [http://www.ncbi.nlm.nih.gov/pubmed/25959774]. For visualizations, smoothed 4C-seq reads were normalized to the 5 TADs surrounding the *Krox20* locus (chr10:62,880,000–70,720,000).

Position of mouse TADs in ES cells and associated 40 kb normalized Hi-C matrices [11] were obtained from http://promoter.bx.psu.edu/hi-c/download.html. Directionality indexes were calculated as described using tools described previously [7]. Interaction matrices are visualized using standard cut-offs.

### ATAC-seq

ATAC experiments were performed according to Buenrostros and colleagues [31], using a homemade transposome [57]. 7–8 embryos at 10-12s were dissected in cold PBS for each genotype and cells were mechanically dissociated. Biological duplicates were performed for ATAC experiments. Cells were lysed before transposition using 1 μl of transposome and purified using a Qiagen MinElute Kit with 10 μl of Elution Buffer. Transposed DNA was amplified by PCR as previously described [57] and quantified by qPCR using 5 μl of PCR products. The number of additional cycles was determined by plotting linear Rn versus cycle and corresponded to one third of the maximum fluorescence intensity. The remaining PCR products (45 μl) were treated with the additional number of cycles. The final product was purified with Qiagen PCR Cleanup Kit and eluted in 20 μl Elution Buffer. Sequencing was performed on multiplexed samples using 42 bp paired-end reads on an Illumina NextSeq according to the manufacturer’s specifications. For computational analysis, paired-end reads were mapped onto the mouse genome assembly mm9, using STAR (outFilterMultimapNmax 1; outFilterMismatchNmax 999; outFilterMismatchNoverLmax 0.06; alignIntronMax 1; alignEndsType EndToEnd; alignMatesGapMax 2000). Duplicates reads were removed using Picard (http://picard.sourceforge.net) (MarkDuplicates, REMOVE_DUPLICATES = true). To consider only fragments coming from transcription factors protected DNA (and not from nucleosomes), only fragment with size lower than 100 bp were kept. Bigwig tracks were obtained using DeepTools BamCoverage (1.5.9.1). Peak calling was performed using MACS2 (2.1.0.20140616), using a q-value< = 0.01 threshold (other parameters as default). For quantification, we first defined a set of non-redundant enriched regions for all samples by taking the union of all peak summits from both replicates of all samples, grouped together all summits distant from less than 50 bp, and for each group kept only the summit with the lowest q-value (calculated by MACS2). We then quantified the signal at all summits in each sample by counting the number of fragments (using the R bioconductor package csaw, v. 1.0.7). Normalisation and statistical analysis were performed using the bioconductor DESeq2 package (1.6.3). Library size factors were calculated on fragment counts in genomic bins of 10 kb. Comparison between wild type, *Krox20*^*ΔC/ΔC*^ and *Krox20*^*ΔA/ΔA*^ embryos was performed using negative binomial Wald Test (DESeq2).

### Accession codes

The data have been deposited in the Gene Expression Omnibus (GEO) under accession number GSE94716 and is available at the following address: https://www.ncbi.nlm.nih.gov/geo/query/acc.cgi?acc=GSE94716

## Supporting information

S1 Fig Element C is required for normal development of the r3 territory. **(A)** Sequence of the deleted element C and associated genomic coordinates (mouse genome assembly mm9). This sequence corresponds to the cloned sequence of the mouse element C described in [13]. **(B)** *In situ* hybridization for *EphA4* mRNA performed on control (*Krox20*^*+/ΔC*^) and homozygous (*Krox20*^*ΔC/ΔC*^) mouse embryos at the indicated somite stages. Note the reduction of the size of r3 in the homozygous mutant. Rhombomeres positions are indicated on the left of each embryo.
(TIF)

S2 Fig The Krox20 regulatory neighborhood includes its regulatory elements but not the *Nrbf2* gene. **(A)** The left boundary of the *Krox20* TAD, as previously determined from Hi-C in ES cells [11], spreads out over a transition zone with a low Directionality Index of Hi-C interactions in both ES cells and adult cortex ([11]). Hi-C in ES cells and TADs as defined in [11] or by our additional analysis (‘with CTCF’, this figure) are indicated above. Dashed lines in the graphs demarcate the boundaries between the -2, -1, *Krox20* and +1 TADs. The transition zone between the -1 and *Krox20* TADs is highlighted in grey. CTCF ChIP-seq data in E14.5 mouse brain (ENCODE, [58]) and genes are indicated below. Orange bars and gene names above pinpoint 4C-seq viewpoints. **(B)** Coordinates of the different regions used for the analysis of the distribution of 4C-seq signal. **(C)** Normalized distribution of 4C-seq signal for the *Nrbf2*, Element A and *Krox20* viewpoints using previously determined TAD boundaries ([11], left) or after the repositioning of the TAD boundary to the cluster of CTCF binding sites between the *Nrbf2* gene and Element A (right). The ratio of signal between the -1 and *Krox20* TADs (average of 2 replicates) is shown above for each viewpoint. When the previously determined TAD boundary is used (left), the 4C-seq signal of the *Nrbf2* viewpoint is almost equally distributed over the -1 and *Krox20* TADs, whereas a much more discrete distribution is observed when the cluster of CTCF sites is used (right). In contrast, the nearby Element A viewpoint always restricts its strongest signal to the *Krox 20* TAD, similar to the associated but much more distant *Krox20* gene.
(TIF)

S3 Fig Chromatin state and accessibility within the *Krox20* locus. **(A-C)** ChIP-seq was performed for the H3K27ac mark on wild type E8.5 embryo heads (light blue) in duplicates and only one replicate is shown. ATAC-seq was performed on dissected regions (r3, r5 and a more posterior region (“post”; see text) from wild type (light blue), *Krox20*^*ΔC/ΔC*^ (green) and *Krox20*^*ΔA/ΔA*^ (red) E8.5 embryos. Genes, *cis*-regulatory elements (orange) and a genomic scale are indicated at the top. CTCF ChIP-seq data in E14.5 mouse brain (ENCODE) are indicated below each panel.
(TIF)

S4 Fig Dynamics of enhancer activity of the new mouse element (NE) in zebrafish. A zebrafish transgenic line *Tg(NE*:*gfp)*, carrying a construct in which NE is linked to the gfp gene driven by a minimal promoter, was analysed by double ISH with *krox20* (orange) and *gfp* (purple) probes at 3s and 10s stages.
(TIF)

S1 Table Sequences of 4C-seq primers including Illumina adaptors. (PDF)

S1 File Supplementary methods. (PDF)

## References

[1] Banerji J, Rusconi S, Schaffner W. Expression of a beta-globin gene is enhanced by remote SV40 DNA sequences. Cell. 1981;27(2 Pt 1):299–308. 6277502 10.1016/0092-8674(81)90413-x

[2] Maniatis T, Goodbourn S, Fischer J a. Regulation of inducible and tissue-specific gene expression. Science. 1987 6 5;236(4806):1237–45. 3296191 10.1126/science.3296191

[3] Long HK, Prescott SL, Wysocka J. Review Ever-Changing Landscapes: Transcriptional Enhancers in Development and Evolution. Cell. 2016;167(5):1170–87. doi: 10.1016/j.cell.2016.09.018 27863239 10.1016/j.cell.2016.09.018 PMC5123704

[4] Kvon EZ. Using transgenic reporter assays to functionally characterize enhancers in animals. Genomics. 2015;106(3):185–92. doi: 10.1016/j.ygeno.2015.06.007 26072435 10.1016/j.ygeno.2015.06.007

[5] Ruf S, Symmons O, Uslu VV, Dolle D, Hot C, Ettwiller L, et al Large-scale analysis of the regulatory architecture of the mouse genome with a transposon-associated sensor. Nat Genet. 2011 4;43(4):379–86. doi: 10.1038/ng.790 21423180 10.1038/ng.790

[6] Symmons O, Uslu VV, Tsujimura T, Ruf S, Nassari S, Schwarzer W, et al Functional and topological characteristics of mammalian regulatory domains. Genome Res. 2014;24(3):390–400. doi: 10.1101/gr.163519.113 24398455 10.1101/gr.163519.113 PMC3941104

[7] Dunipace L, Saunders A, Ashe H, Stathopoulos A. Autoregulatory feedback controls sequential action of cis-regulatory modules at the brinker locus. Dev Cell. 2013;26(5):536–43. doi: 10.1016/j.devcel.2013.08.010 24044892 10.1016/j.devcel.2013.08.010 PMC3782659

[8] Zaret KS, Carroll JS. Pioneer transcription factors: Establishing competence for gene expression. Genes Dev. 2011;25(21):2227–41. doi: 10.1101/gad.176826.111 22056668 10.1101/gad.176826.111 PMC3219227

[9] Gibcus JH, Dekker J. The hierarchy of the 3D genome. Mol Cell. 2013 3 7;49(5):773–82. doi: 10.1016/j.molcel.2013.02.011 23473598 10.1016/j.molcel.2013.02.011 PMC3741673

[10] Nora EP, Lajoie BR, Schulz EG, Giorgetti L, Okamoto I, Servant N, et al Spatial partitioning of the regulatory landscape of the X-inactivation centre. Nature. 2012 5 17;485(7398):381–5. doi: 10.1038/nature11049 22495304 10.1038/nature11049 PMC3555144

[11] Dixon JR, Selvaraj S, Yue F, Kim A, Li Y, Shen Y, et al Topological domains in mammalian genomes identified by analysis of chromatin interactions. Nature. 2012 5 17;485(7398):376–80. doi: 10.1038/nature11082 22495300 10.1038/nature11082 PMC3356448

[12] Chavrier P, Zerial M, Lemaire P, Almendral J, Bravo R, Charnay P. A gene encoding a protein with zinc fingers is activated during G0/G1 transition in cultured cells. EMBO J. 1988;7(1):29–35. 3129290 10.1002/j.1460-2075.1988.tb02780.x PMC454212

[13] Chomette D, Frain M, Cereghini S, Charnay P, Ghislain J. Krox20 hindbrain cis-regulatory landscape: interplay between multiple long-range initiation and autoregulatory elements. Development. 2006 4;133(7):1253–62. doi: 10.1242/dev.02289 16495311 10.1242/dev.02289

[14] Lumsden A, Krumlauf R. Patterning the vertebrate neuraxis. Science. 1996 11 15;274(5290):1109–15. 8895453 10.1126/science.274.5290.1109

[15] Schneider-Maunoury S, Topilko P, Seitandou T, Levi G, Cohen-Tannoudji M, Pournin S, et al Disruption of Krox-20 results in alteration of rhombomeres 3 and 5 in the developing hindbrain. Cell. 1993 12 17;75(6):1199–214. 7903221 10.1016/0092-8674(93)90329-o

[16] Schneider-Maunoury S, Seitanidou T, Charnay P, Lumsden a. Segmental and neuronal architecture of the hindbrain of Krox-20 mouse mutants. Development. 1997 3;124(6):1215–26. 9102308 10.1242/dev.124.6.1215

[17] Voiculescu O, Taillebourg E, Pujades C, Kress C, Buart S, Charnay P, et al Hindbrain patterning: Krox20 couples segmentation and specification of regional identity. Development. 2001 12;128(24):4967–78. 11748134 10.1242/dev.128.24.4967

[18] Bouchoucha YX, Reingruber J, Labalette C, Wassef M a, Thierion E, Desmarquet-Trin Dinh C, et al Dissection of a Krox20 positive feedback loop driving cell fate choices in hindbrain patterning. Mol Syst Biol. 2013 9 24;9(690). 10.1038/msb.2013.46 PMC3792346 24061538

[19] Labalette C, Bouchoucha YX, Wassef MA, Gongal PA, Le Men J, Becker T, et al Hindbrain patterning requires fine-tuning of early krox20 transcription by Sprouty 4. Development. 2011 1;138(2):317–26. doi: 10.1242/dev.057299 21177344 10.1242/dev.057299

[20] Labalette C, Wassef MA, Desmarquet-Trin Dinh C, Bouchoucha YX, Le Men J, Charnay P, et al Molecular dissection of segment formation in the developing hindbrain. Development. 2015 1 1;142(1):185–95. doi: 10.1242/dev.109652 25516974 10.1242/dev.109652

[21] Wassef M a, Chomette D, Pouilhe M, Stedman A, Havis E, Desmarquet-Trin Dinh C, et al Rostral hindbrain patterning involves the direct activation of a Krox20 transcriptional enhancer by Hox/Pbx and Meis factors. Development. 2008 10;135(20):3369–78. doi: 10.1242/dev.023614 18787068 10.1242/dev.023614

[22] Seitanidou T, Schneider-Maunoury S, Desmarquet C, Wilkinson DG, Charnay P. Krox-20 is a key regulator of rhombomere-specific gene expression in the developing hindbrain. Mech Dev. 1997 7;65(1–2):31–42. 9256343 10.1016/s0925-4773(97)00051-8

[23] Voiculescu O, Charnay P, Schneider-Maunoury S. Expression pattern of a Krox-20/Cre knock-in allele in the developing hindbrain, bones, and peripheral nervous system. Genesis. 2000 2;26(2):123–6. 10686605 10.1002/(sici)1526-968x(200002)26:2<123::aid-gene7>3.0.co;2-o

[24] van de Werken HJG, Landan G, Holwerda SJB, Hoichman M, Klous P, Chachik R, et al Robust 4C-seq data analysis to screen for regulatory DNA interactions. Nat Methods. 2012;9(10):969–72. doi: 10.1038/nmeth.2173 22961246 10.1038/nmeth.2173

[25] Irving C, Nieto M a, DasGupta R, Charnay P, Wilkinson DG. Progressive spatial restriction of Sek-1 and Krox-20 gene expression during hindbrain segmentation. Dev Biol. 1996;173(1):26–38. doi: 10.1006/dbio.1996.0004 8575627 10.1006/dbio.1996.0004

[26] Barski A, Cuddapah S, Cui K, Roh TY, Schones DE, Wang Z, et al High-Resolution Profiling of Histone Methylations in the Human Genome. Cell. 2007;129(4):823–37. doi: 10.1016/j.cell.2007.05.009 17512414 10.1016/j.cell.2007.05.009

[27] Zhou VW, Goren A, Bernstein BE. Charting histone modifications and the functional organization of mammalian genomes. Nat Rev Genet. 2011 1;12(1):7–18. doi: 10.1038/nrg2905 21116306 10.1038/nrg2905

[28] Ghislain J, Desmarquet-Trin-Dinh C, Gilardi-Hebenstreit P, Charnay P, Frain M. Neural crest patterning: autoregulatory and crest-specific elements co-operate for Krox20 transcriptional control. Development. 2003;130(5):941–53. 12538520 10.1242/dev.00318

[29] Theil T, Frain M, Gilardi-Hebenstreit P, Flenniken A, Charnay P, Wilkinson DG. Segmental expression of the EphA4 (Sek-1) receptor tyrosine kinase in the hindbrain is under direct transcriptional control of Krox-20. Development. 1998 2;125(3):443–52. 9425139 10.1242/dev.125.3.443

[30] Wood HB, Episkopou V. Comparative expression of the mouse Sox1, Sox2 and Sox3 genes from pre-gastrulation to early somite stages. Mech Dev. 1999;86(1–2):197–201. 10446282 10.1016/s0925-4773(99)00116-1

[31] Buenrostro JD, Giresi PG, Zaba LC, Chang HY, Greenleaf WJ. Transposition of native chromatin for fast and sensitive epigenomic profiling of open chromatin, DNA-binding proteins and nucleosome position. Nat Methods. 2013;10(12):1213–8. doi: 10.1038/nmeth.2688 24097267 10.1038/nmeth.2688 PMC3959825

[32] Labalette C, Wassef MA, Desmarquet-Trin Dinh C, Bouchoucha YX, Le Men J, Charnay P, et al Molecular dissection of segment formation in the developing hindbrain. Development. 2015;142(1):185–95. doi: 10.1242/dev.109652 25516974 10.1242/dev.109652

[33] Wiellette EL, Sive H. vhnf1 and Fgf signals synergize to specify rhombomere identity in the zebrafish hindbrain. Development. 2003;130(16):3821–9. 12835397 10.1242/dev.00572

[34] Cordes SP, Barsh GS. The mouse segmentation gene kr encodes a novel basic domain-leucine zipper transcription factor. Cell. 1994 12 16;79(6):1025–34. 8001130 10.1016/0092-8674(94)90033-7

[35] Waskiewicz AJ, Rikhof H a, Moens CB. Reveals a Hindbrain Ground State. Dev Cell. 2002;3:723–33. 12431378 10.1016/s1534-5807(02)00319-2

[36] Choe S-K, Vlachakis N, Sagerström CG. Meis family proteins are required for hindbrain development in the zebrafish. Development. 2002;129(3):585–95. 11830560 10.1242/dev.129.3.585

[37] McNulty CL, Peres JN, Bardine N, van den Akker WMR, Durston AJ. Knockdown of the complete Hox paralogous group 1 leads to dramatic hindbrain and neural crest defects. Development. 2005;132(12):2861–71. doi: 10.1242/dev.01872 15930115 10.1242/dev.01872

[38] Walshe J, Maroon H, McGonnell IM, Dickson C, Mason I. Establishment of hindbrain segmental identity requires signaling by FGF3 and FGF8. Curr Biol. 2002;12(13):1117–23. 12121619 10.1016/s0960-9822(02)00899-0

[39] Aragon F, Pujades C. FGF signaling controls caudal hindbrain specification through Ras-ERK1/2 pathway. BMC Dev Biol. 2009;9:61 doi: 10.1186/1471-213X-9-61 19958530 10.1186/1471-213X-9-61 PMC2794271

[40] Hong J-W, Hendrix D a, Levine MS. Shadow enhancers as a source of evolutionary novelty. Science. 2008;321(5894):1314 doi: 10.1126/science.1160631 18772429 10.1126/science.1160631 PMC4257485

[41] Kurokawa D, Kiyonari H, Nakayama R, Kimura-Yoshida C, Matsuo I, Aizawa S. Regulation of Otx2 expression and its functions in mouse forebrain and midbrain. Development. 2004 7;131(14):3319–31. doi: 10.1242/dev.01220 15201224 10.1242/dev.01220

[42] Lam DD, de Souza FSJ, Nasif S, Yamashita M, López-Leal R, Otero-Corchon V, et al Partially Redundant Enhancers Cooperatively Maintain Mammalian Pomc Expression Above a Critical Functional Threshold. PLOS Genet. 2015;11(2):e1004935 doi: 10.1371/journal.pgen.1004935 25671638 10.1371/journal.pgen.1004935 PMC4335486

[43] Lagha M, Bothma JP, Levine M. Mechanisms of transcriptional precision in animal development. Trends Genet. 2012 8;28(8):409–16. doi: 10.1016/j.tig.2012.03.006 22513408 10.1016/j.tig.2012.03.006 PMC4257495

[44] Ludwig MZ, Manu, Kittler R, White KP, Kreitman M. Consequences of eukaryotic enhancer architecture for gene expression dynamics, development, and fitness. PLoS Genet. 2011;7(11). 10.1371/journal.pgen.1002364 PMC3213169 22102826

[45] Leddin M, Perrod C, Hoogenkamp M, Ghani S, Assi S, Heinz S, et al Two distinct auto-regulatory loops operate at the PU.1 locus in B cells and myeloid cells. Blood. 2011;117(10):2827–38. doi: 10.1182/blood-2010-08-302976 21239694 10.1182/blood-2010-08-302976 PMC3062295

[46] Li W, Notani D, Rosenfeld MG. Enhancers as non-coding RNA transcription units: recent insights and future perspectives. Nat Rev Genet. 2016;17(4):207–23. doi: 10.1038/nrg.2016.4 26948815 10.1038/nrg.2016.4

[47] Lallemand Y, Luria V, Haffner-Krausz R, Lonai P. Maternally expressed PGK-Cre transgene as a tool for early and uniform activation of the Cre site-specific recombinase. Transgenic Res. 1998;7(2):105–12. 9608738 10.1023/a:1008868325009

[48] Giudicelli F, Taillebourg E, Charnay P, Gilardi-Hebenstreit P. Krox-20 patterns the hindbrain through both cell-autonomous and non cell-autonomous mechanisms. Genes Dev. 2001 3 1;15(5):567–80. doi: 10.1101/gad.189801 11238377 10.1101/gad.189801 PMC312642

[49] Wilkinson DG, Bhatt S, Chavrier P, Bravo R, Charnay P. Segment-specific expression of a zinc-finger gene in the developing nervous system of the mouse. Nature. 1989 2 2;337(6206):461–4. doi: 10.1038/337461a0 2915691 10.1038/337461a0

[50] Gilardi-Hebenstreit P, Nieto MA, Frain M, Mattéi MG, Chestier A, Wilkinson DG, et al An Eph-related receptor protein tyrosine kinase gene segmentally expressed in the developing mouse hindbrain. Oncogene. 1992 12;7(12):2499–506. 1281307

[51] Vitobello A, Ferretti E, Lampe X, Vilain N, Ducret S, Ori M, et al Hox and Pbx Factors Control Retinoic Acid Synthesis during Hindbrain Segmentation. Dev Cell. 2011;20(4):469–82. doi: 10.1016/j.devcel.2011.03.011 21497760 10.1016/j.devcel.2011.03.011 PMC3677862

[52] Jourdren L, Bernard M, Dillies MA, Le Crom S. Eoulsan: A cloud computing-based framework facilitating high throughput sequencing analyses. Bioinformatics. 2012;28(11):1542–3. doi: 10.1093/bioinformatics/bts165 22492314 10.1093/bioinformatics/bts165

[53] Dobin A, Davis CA, Schlesinger F, Drenkow J, Zaleski C, Jha S, et al STAR: Ultrafast universal RNA-seq aligner. Bioinformatics. 2013;29(1):15–21. doi: 10.1093/bioinformatics/bts635 23104886 10.1093/bioinformatics/bts635 PMC3530905

[54] Ramírez F, Dündar F, Diehl S, Grüning BA, Manke T. DeepTools: A flexible platform for exploring deep-sequencing data. Nucleic Acids Res. 2014;42(W1):187–91. 10.1093/nar/gku365 PMC4086134 24799436

[55] Matelot M, Noordermeer D. Determination of High-Resolution 3D Chromatin Organization Using Circular Chromosome Conformation Capture (4C-seq). 2016;223–41. 10.1007/978-1-4939-6380-5_20 27659989

[56] David FPA, Delafontaine J, Carat S, Ross FJ, Lefebvre G, Jarosz Y, et al HTSstation: A web application and open-access libraries for high-throughput sequencing data analysis. PLoS One. 2014;9(1). 10.1371/journal.pone.0085879 PMC3903476 24475057

[57] Picelli S, Björklund AK, Reinius B, Sagasser S, Winberg G, Sandberg R. Tn5 transposase and tagmentation procedures for massively scaled sequencing projects. Genome Res. 2014;24(12):2033–40. doi: 10.1101/gr.177881.114 25079858 10.1101/gr.177881.114 PMC4248319

[58] Shen Y, Yue F, McCleary DF, Ye Z, Edsall L, Kuan S, et al A map of the cis-regulatory sequences in the mouse genome. Nature. 2012;488(7409):116–20. doi: 10.1038/nature11243 22763441 10.1038/nature11243 PMC4041622

