# Supplementary figures and images for: Krox20 hindbrain regulation incorporates multiple modes of cooperation between cis-acting elements
Source: PLoS Genet. 2017 Jul 27;13(7):e1006903. doi: 10.1371/journal.pgen.1006903 (PMC5549768; doi:10.1371/journal.pgen.1006903)

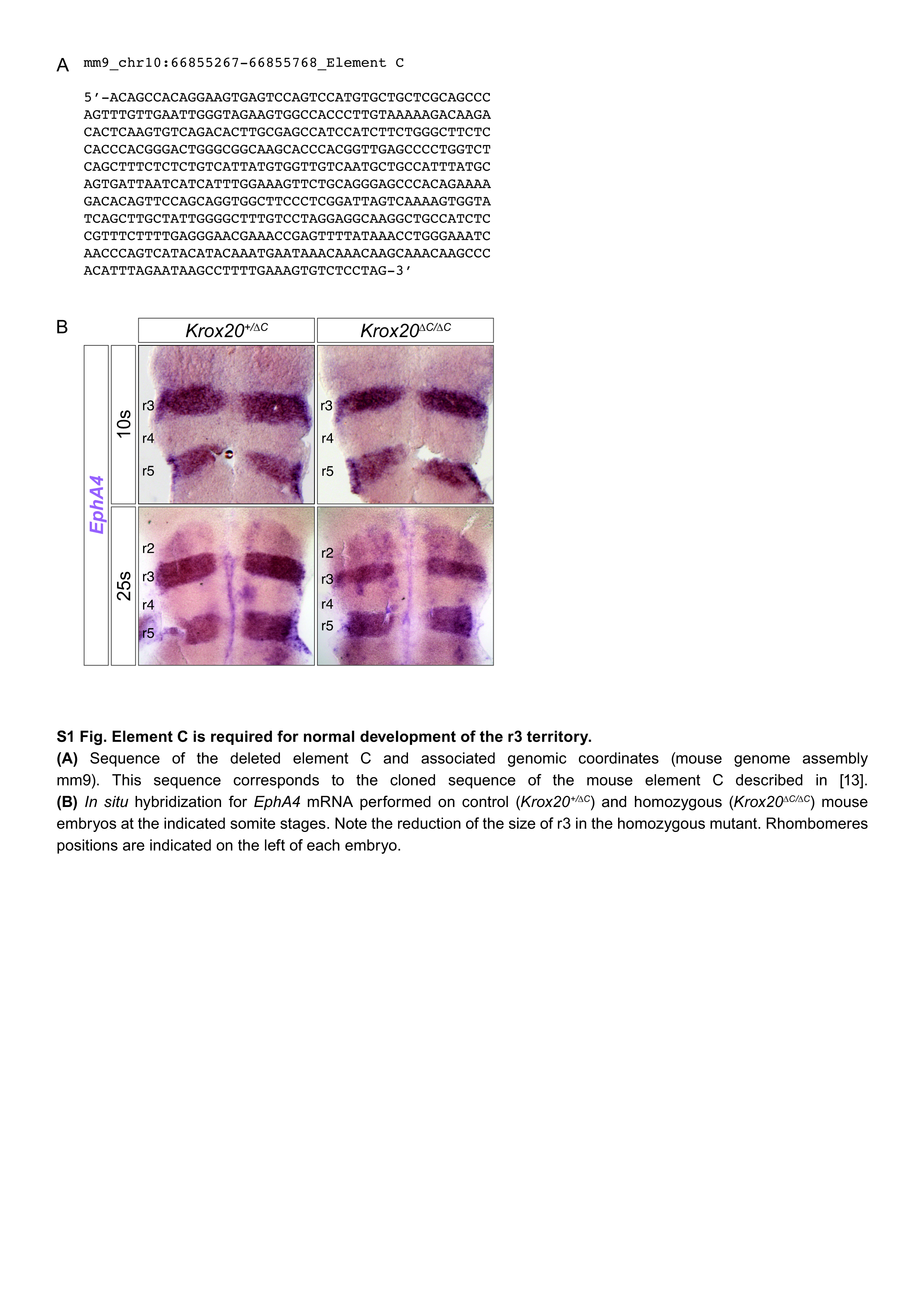

Supplement: S1 Fig — (A) Sequence of the deleted element C and associated genomic coordinates (mouse genome assembly mm9). This sequence corresponds to the cloned sequence of the mouse element C described in [13]. (B) In situ hybridization for EphA4 mRNA performed on control (Krox20+/ΔC) and homozygous (Krox20ΔC/ΔC) mouse embryos at the indicated somite stages. Note the reduction of the size of r3 in the homozygous mutant. Rhombomeres positions are indicated on the left of each embryo. (TIF) [file pgen.1006903.s001.tif]

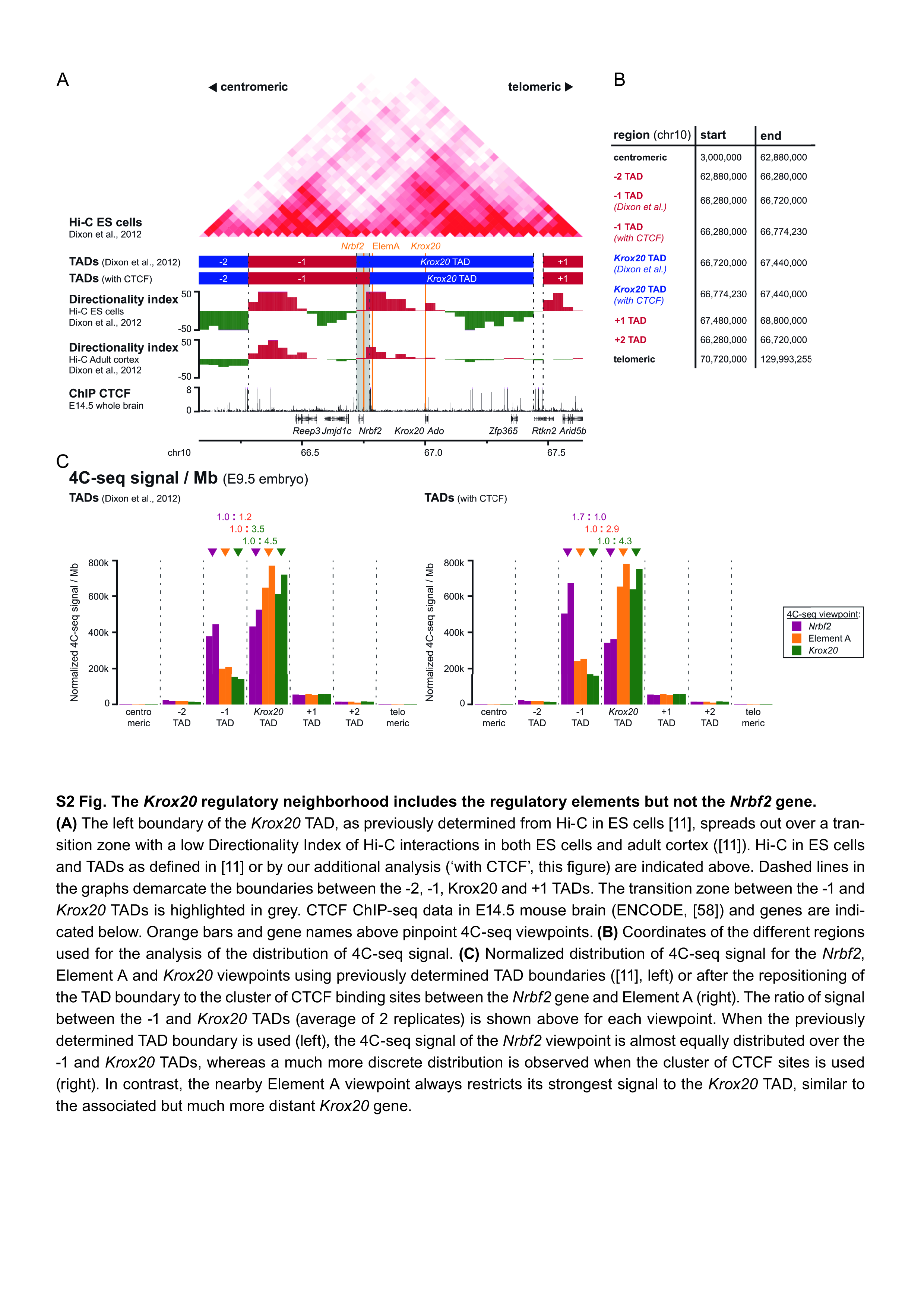

Supplement: S2 Fig — (A) The left boundary of the Krox20 TAD, as previously determined from Hi-C in ES cells [11], spreads out over a transition zone with a low Directionality Index of Hi-C interactions in both ES cells and adult cortex ([11]). Hi-C in ES cells and TADs as defined in [11] or by our additional analysis (‘with CTCF’, this figure) are indicated above. Dashed lines in the graphs demarcate the boundaries between the -2, -1, Krox20 and +1 TADs. The transition zone between the -1 and Krox20 TADs is highlighted in grey. CTCF ChIP-seq data in E14.5 mouse brain (ENCODE, [58]) and genes are indicated below. Orange bars and gene names above pinpoint 4C-seq viewpoints. (B) Coordinates of the different regions used for the analysis of the distribution of 4C-seq signal. (C) Normalized distribution of 4C-seq signal for the Nrbf2, Element A and Krox20 viewpoints using previously determined TAD boundaries ([11], left) or after the repositioning of the TAD boundary to the cluster of CTCF binding sites between the Nrbf2 gene and Element A (right). The ratio of signal between the -1 and Krox20 TADs (average of 2 replicates) is shown above for each viewpoint. When the previously determined TAD boundary is used (left), the 4C-seq signal of the Nrbf2 viewpoint is almost equally distributed over the -1 and Krox20 TADs, whereas a much more discrete distribution is observed when the cluster of CTCF sites is used (right). In contrast, the nearby Element A viewpoint always restricts its strongest signal to the Krox 20 TAD, similar to the associated but much more distant Krox20 gene. (TIF) [file pgen.1006903.s002.tif]

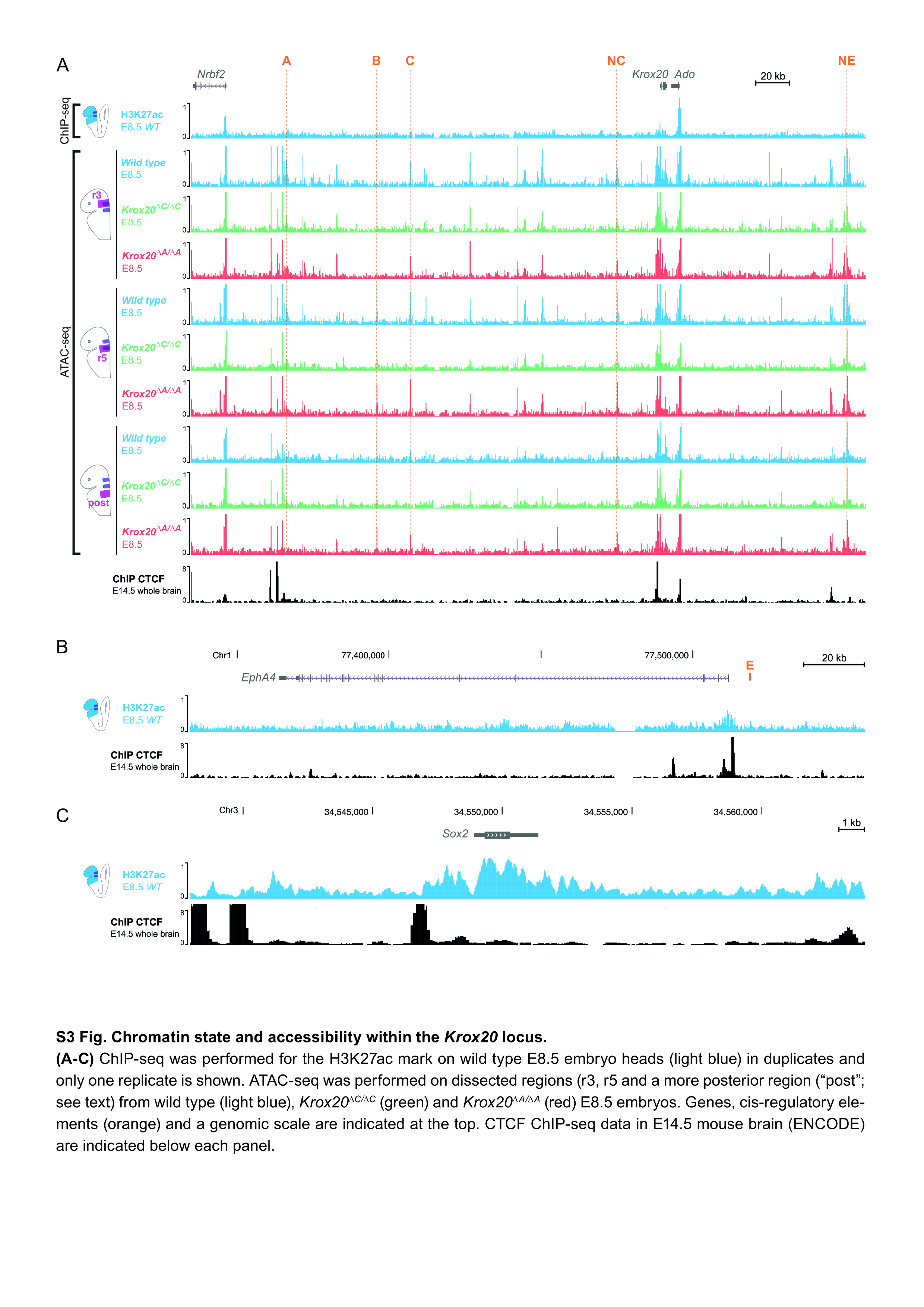

Supplement: S3 Fig — (A-C) ChIP-seq was performed for the H3K27ac mark on wild type E8.5 embryo heads (light blue) in duplicates and only one replicate is shown. ATAC-seq was performed on dissected regions (r3, r5 and a more posterior region (“post”; see text) from wild type (light blue), Krox20ΔC/ΔC (green) and Krox20ΔA/ΔA (red) E8.5 embryos. Genes, cis-regulatory elements (orange) and a genomic scale are indicated at the top. CTCF ChIP-seq data in E14.5 mouse brain (ENCODE) are indicated below each panel. (TIF) [file pgen.1006903.s003.tif]

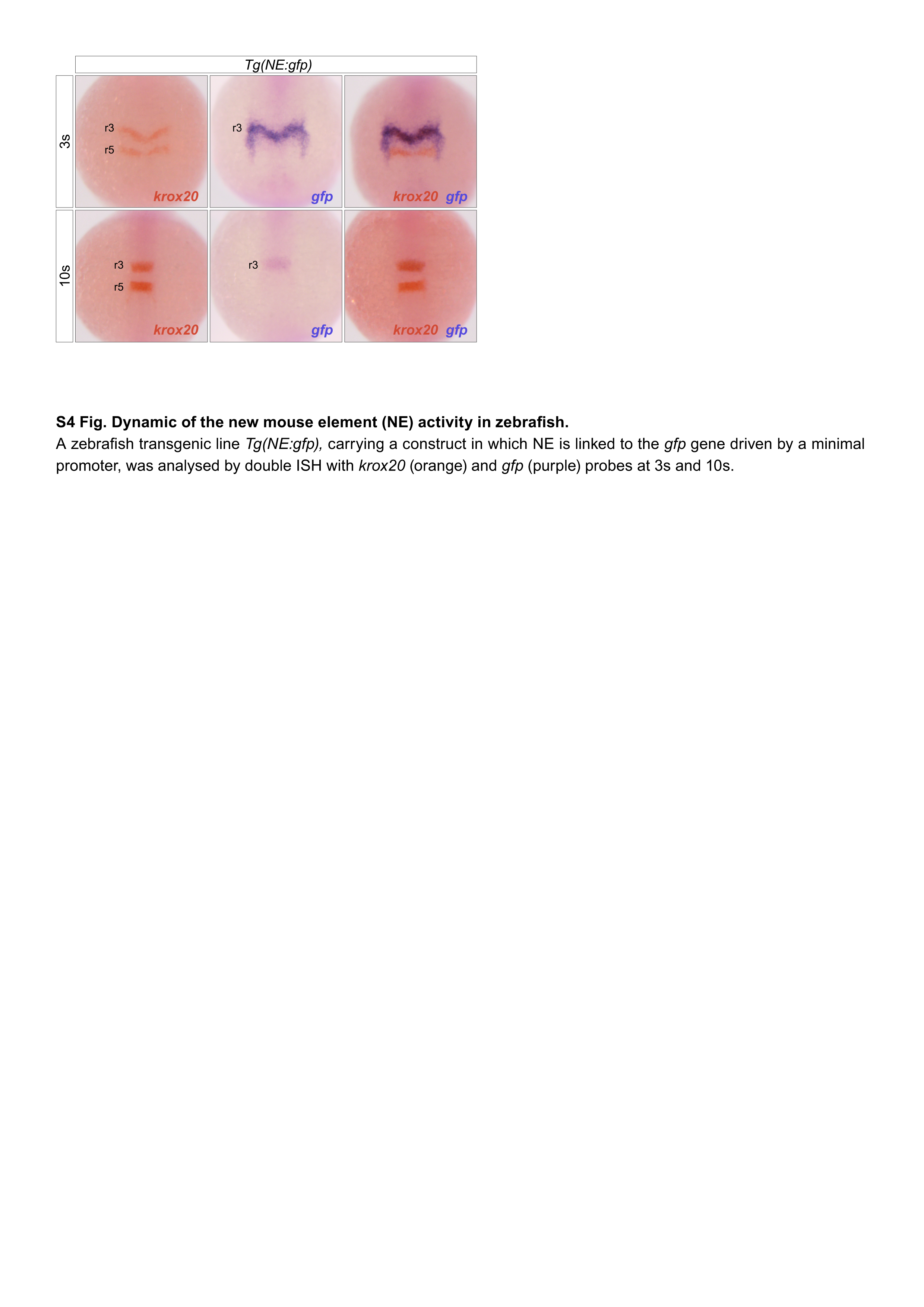

Supplement: S4 Fig — A zebrafish transgenic line Tg(NE:gfp), carrying a construct in which NE is linked to the gfp gene driven by a minimal promoter, was analysed by double ISH with krox20 (orange) and gfp (purple) probes at 3s and 10s stages. (TIF) [file pgen.1006903.s004.tif]
