# Supplementary material for: Krox20 hindbrain regulation incorporates multiple modes of cooperation between cis-acting elements
Source: PLoS Genet. 2017 Jul 27;13(7):e1006903. doi: 10.1371/journal.pgen.1006903 (PMC5549768; doi:10.1371/journal.pgen.1006903)
Supplement: S1 Table — (PDF) [file pgen.1006903.s005.pdf]

S1 Table. Sequences of 4C-seq primers including Illumina adaptors

Primer names	Sequences
Krox20 promoter	Inverse Forward:<br>AATGATACGGCGACCACCGAACACTCTTTCCCTACACGACGCTCT<br>TCCGATCTTCCGTCACTGACTCTCTC<br>Inverse Reverse:<br>CAAGCAGAAGACGGCATACGAAGATGCACCTGGTCACCA
Element A	Inverse Forward:<br>AATGATACGGCGACCACCGAACACTCTTTCCCTACACGACGCTCT<br>TCCGATCTGAGGTAGGGAGAAACAGTAG<br>Inverse Reverse:<br>CAAGCAGAAGACGGCATACGATGAGTCTTGGCAGCTAATG
Element B	Inverse Forward:<br>AATGATACGGCGACCACCGAACACTCTTTCCCTACACGACGCTCT<br>TCCGATCTCTTTGTTCCTTTTGCTCAGT<br>Inverse Reverse:<br>CAAGCAGAAGACGGCATACGAGATGTGACAGAGGTTGAGAG
Element C	Inverse Forward:<br>AATGATACGGCGACCACCGAACACTCTTTCCCTACACGACGCTCT<br>CCGATCTATTTGAAAAATGATGTATCACTGAG<br>Inverse Reverse:<br>CAAGCAGAAGACGGCATACGACTGACAGCCACAGGAAGT
Nrbf2 promoter	Inverse Forward:<br>AATGATACGGCGACCACCGAACACTCTTTCCCTACACGACGCTCT<br>TCCGATCTCACATACTAAATAACAAATGCCATAG<br>Inverse Reverse:<br>CAAGCAGAAGACGGCATACGAAGATTATTATACTCAGCTTGGGA
