# Supplementary material for: Krox20 hindbrain regulation incorporates multiple modes of cooperation between cis-acting elements
Source: PLoS Genet. 2017 Jul 27;13(7):e1006903. doi: 10.1371/journal.pgen.1006903 (PMC5549768; doi:10.1371/journal.pgen.1006903)
Supplement: S1 File — (PDF) [file pgen.1006903.s006.pdf]

S1 File. Supplementary methods.

Zebrafish lines and egg injection

All animal experimentations were performed according to French and European regulations (French agreement: #848 – 2015061510065446 v3). Transgenic lines were obtained from embryos injected at the one-cell stage with a pZED construct, together with 50 pg of tol2 transposase mRNA [1].

Cloning

The mouse NE element was cloned into the pZED plasmid [2], upstream of the minimal *gata2* promoter/*gfp* reporter, following PCR amplification using the following primers: 5'- GCAAACTGGAGGTCAGGTCC and 5'- GGGAGAAAAGGACAACTGTGTC.

In situ hybridization

Single and double whole-mount ISHs were performed as described [3], using the following probes: *krox20* [4] and *gfp* [5].

References:

[1] Kawakami K, Takeda H, Kawakami N, Kobayashi M, Matsuda N, Mishina M. A transposon-mediated gene trap approach identifies developmentally regulated genes in zebrafish. Dev Cell 2004;7:133–44. doi:10.1016/j.devcel.2004.06.005.

[2] Bessa J, Tena JJ, De La Calle-Mustienes E, Fernández-Miñán A, Naranjo S, Fernández A, et al. Zebrafish Enhancer Detection (ZED) vector: A new tool to facilitate transgenesis and the functional analysis of cis-regulatory regions in zebrafish. Dev Dyn 2009;238:2409–17. doi:10.1002/dvdy.22051.

[3] Hauptmann G, Gerster T. Two-color whole-mount in situ hybridization to vertebrate and Drosophila embryos. Trends Genet 1994;10:266.

[4] Zuchegna C, Aceto F, Bertoni A, Romano A, Perillo B, Laccetti P, et al. NAR Breakthrough Article Mechanism of retinoic acid-induced transcription : histone code , DNA oxidation and formation of chromatin loops 2014;42:11040–55.

doi:10.1093/nar/gku823.

[5] Labalette C, Wassef MA, Desmarquet-Trin Dinh C, Bouchoucha YX, Le Men J, Charnay P, et al. Molecular dissection of segment formation in the developing hindbrain. Development 2015;142:185–95. doi:10.1242/dev.109652.
